# Vision Rehabilitation is Part of AMD Care

**DOI:** 10.3390/vision2010004

**Published:** 2018-01-25

**Authors:** August Colenbrander

**Affiliations:** The Smith-Kettlewell Eye Research Institute, San Francisco, CA 94115-1813, USA; gus@ski.org

**Keywords:** vision rehabilitation, low vision, AMD, age-related macular degeneration, detail vision, visual acuity, surround vision, visual field, contrast vision

## Abstract

AMD does not just affect the retina. It severely affects people’s lives. Paying attention to this aspect will only become more important as the population ages and more otherwise healthy individuals become affected. This paper will discuss the need for teamwork to overcome the difference between medical care, which addresses the *causes* of AMD, and rehabilitative care, which addresses the *consequences*. Different aspects and different degrees of vision loss ask for different interventions. Loss of detailed vision can be addressed by a wide variety of magnification devices. The means to address this aspect are well recognized. Surround vision is largely processed pre-attentively. Its loss cannot be remediated by devices, but must be addressed through patient education to bring previously subconscious reactions to conscious awareness. Loss of contrast vision is an aspect that is not sufficiently studied. It is important for early detection, and for the safety of the patient. When the eye condition cannot be modified, environmental modifications provide the most effective remediation.

## 1. Introduction

Most of this issue discusses age-related macular degeneration from the point of view of the “eye” doctor, who is in charge of addressing its causes. This paper wants to draw attention to the point of view of the patient, who needs to deal with its consequences. How can we improve the quality of life of the patient, even if “nothing more can be done” about the condition of the retina?

### 1.1. Patient-Centered Functional Priorities

When considering how various degrees of vision loss may impact a person’s life, the American Academy of Ophthalmology asks for attention for the following five aspects [1].

- **Reading**—many patients express this as their foremost concern.
- **Activities of Daily Living**—even though reading may be the most prominent complaint, most people spend a larger part of their day performing a variety of other vision-dependent activities, such as self-care and home management.
- **Safety**—are people at risk for falls? How do they cross the street? Do they drive?
- **Community participation**—can the individual still participate in community events, including religious gatherings?
- **Physical, cognitive and psychosocial well-being**—since many patients with vision loss are elderly, these are important aspects that should not be overlooked. If cognitive problems exist, it may affect the recommendations to be made.

### 1.2. Four Aspects of Vision Loss

**Figure 1:**
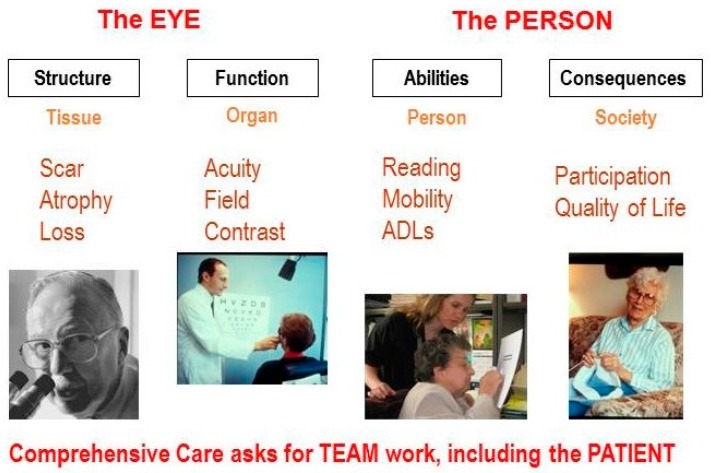
Aspects of visual functioning.

To put this in a broader perspective, I have found it useful to discuss four aspects of visual functioning (Figure 1).

First, we must consider *structural changes*. For this aspect, the focus is on the tissue; an ocular pathologist is needed to describe the pathological condition.

However, structural changes alone do not determine how well the eyes actually function. We need to expand our focus and measure *organ functions*, such as visual acuity, visual field, and contrast sensitivity.

Yet, even knowing how the eyes function does not tell us how the person functions. We must further expand our focus to the person, and to the *ability* to perform tasks, such as reading, mobility, face recognition and activities of daily living (ADL). Here we need the help of occupational therapists and other rehabilitation professionals.

Finally, we must assess the consequences for the person in a societal context. Does the vision loss have an impact on the person’s *participation in society*, and on general satisfaction with one’s quality of life? It is clear that no single professional can cover all aspects. A team approach is necessary, and the patient must be part of that team [2].

It is helpful to draw a line between the organ side of Figure 1 on the left, and the person side on the right. On the left side of the diagram, we discuss visual functions, such as visual acuity and visual field. Eye doctors are interested in these aspects, which describe *How the EYES function*, since their task is to find the underlying causes of eye disease. On the right side, we speak of functional vision, which describes *How the PERSON functions* when performing vision-related activities. Here, the goal is to address the societal consequences of eye disease. These different objectives explain why the perspectives of the doctor and the patient differ.

### 1.3. Vision Rehabilitation

**Figure 2:**
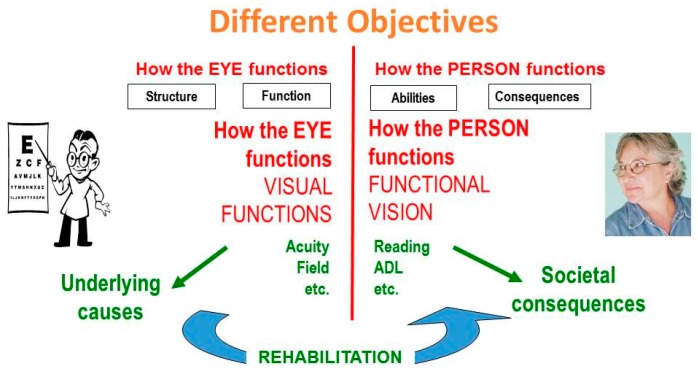
The role of rehabilitation.

Vision rehabilitation is tasked with bridging the gap. “Eye” doctors should not restrict their attention to the eye; they should become “people” doctors by extending their interest to the right side of the diagram as well (Figure 2). The different objectives also ask for different interventions. On the doctor’s side are medical and surgical interventions; on the other side are rehabilitative interventions.

**Figure 3:**
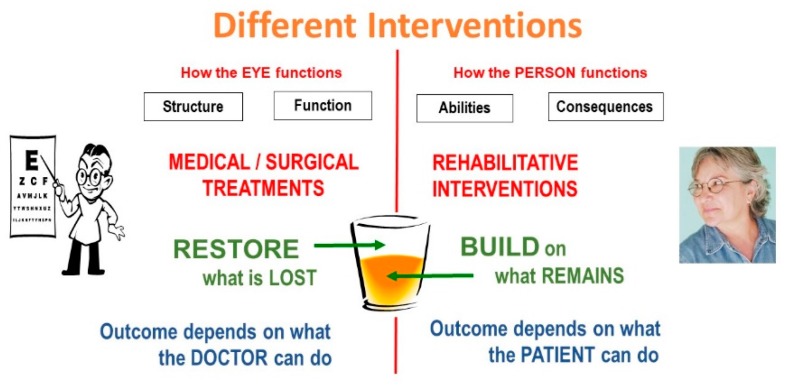
Different interventions.

Medical treatments aim at restoring what was lost. They deal with the part of the glass that is half empty. The outcome depends on the tool kit and skills of the physician. Rehabilitative interventions, on the other hand, build on what remains in the part of the glass that is half full. Their outcome depends on what the patient can do with the help of our guidance. We can give the patient crutches, the patient must do the walking. We can give the patient a magnifier, the patient must do the reading (Figure 3).

**Figure 4:**
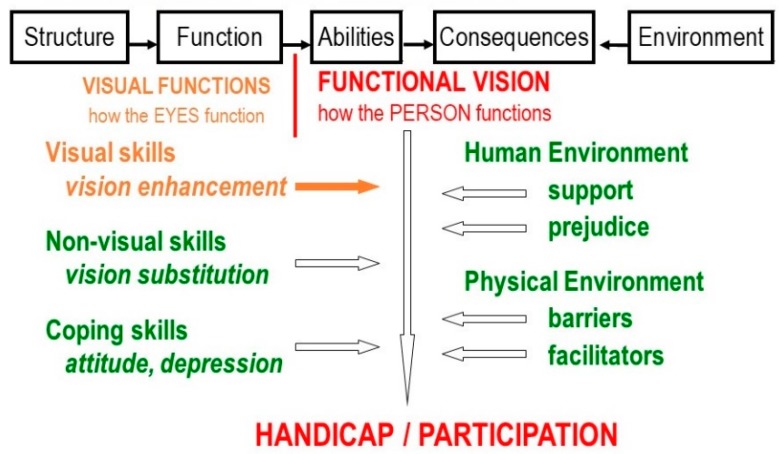
Factors in comprehensive vision rehabilitation.

Comprehensive rehabilitation, therefore, must consider many aspects beyond the eye and even beyond vision (Figure 4).

At this point, many specialists may feel that this comprehensive approach involves more than what they were trained for, or than they are prepared to take on. That is why a team approach is necessary. Most clinicians are comfortable referring various simple activities to technicians in their office and have no problem referring difficult cases to another specialist. The same should be true for vision rehabilitation. The difference between vision rehabilitation and other sub-specialties is that most ophthalmic sub-specialties are anatomically defined; vision rehabilitation is functionally defined and therefore cuts across all other sub-specialties.

In a group practice, simple procedures, such as having the patient try a magnifier or prescribing a higher reading add for early macular degeneration, can be handled by ancillary personnel in the office. More involved cases may require an outside referral.

It is essential, however, that the treating physician makes the referral and takes credit for it. Referral should not wait until the patient has severe complaints. Anticipating the patient’s needs and making appropriate referrals will result in happier patients. The smartsight program of the AAO recommends that rehabilitation needs should be considered for any patient with less than 20/40 acuity [3].

**Figure 5:**
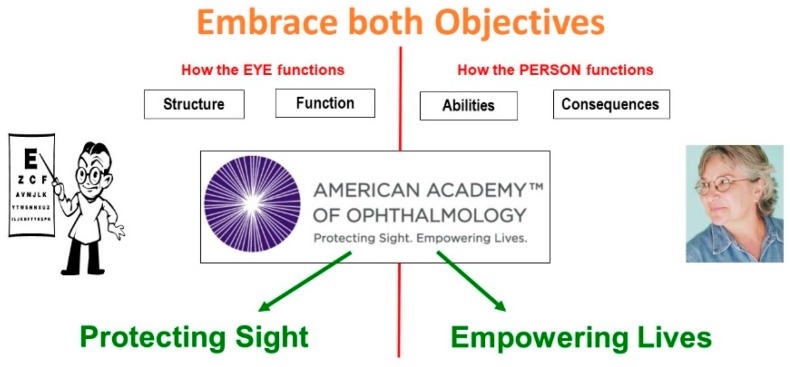
Factors in comprehensive vision rehabilitation.

The motto of the American Academy of Ophthalmology expresses the dual objective of attending to the eye as well as to the person by including two equally important objectives: Protecting sight, as well as Empowering lives (Figure 5).

### 1.4. Functional Assessment

After determining the anatomical and structural condition of the eye, attention must also be given to the functioning of the eye and of the patient. The most important aspects in relation to AMD are:

- **Detail vision**—Usually measured as visual acuity.
  Letter chart acuity describes only the function of the central 1-degree area.
- **Surround vision**—Usually described as visual field
  Surround vision involves everything beyond the central 1-degree area.
- **Contrast vision**—Interacts with both acuity and field.
  Better illumination and better contrast can result in better acuity and a better field.

There are other aspects of vision, such as color vision, depth perception, motion detection, and others, but they have a less direct impact on the overall functioning of the patient.

We use the terms detail vision, surround vision and contrast vision to stress that they each represent only one aspect of vision and to avoid the common overly broad interpretation of the term “visual acuity” as if it were equivalent to vision in general. Detail vision is only one aspect of vision, and visual acuity is only one of the scales on which it can be measured. Retinal specialists may be more used to the term central vision, but the adjective central refers only to retinal topography. Detail vision refers to the purpose for which this component of vision is used; in the context of rehabilitation, the purpose is more relevant than the topography.

We will discuss the assessment and remediation of problems in each of the three areas mentioned above.

## 2. Detail Vision

A reduction in the ability to see details represents the most common visual complaint, usually expressed as difficulty reading. Its assessment is usually done with a letter chart, and the result of that measurement is called “visual acuity”. The letter chart as a measurement tool was introduced by Snellen in 1862 [4] and has become so widely used that the term visual acuity is often interpreted as a global descriptor of “vision” in general. This is not correct.

The ability to recognize single letters assesses only the retinal area where each letter is projected. Even for a 20/200 letter (10×), that area is less than one degree. For optical problems (refractive error, opacities), this is no problem, since central defocus predicts similar peripheral defocus. For retinal problems, however, the visual acuity value does not tell us anything about the surrounding retina. So, we also need to assess the area beyond the 1-degree area used for fixation, which will be discussed under surround vision.

### 2.1. Recording Detail Vision

Just as distances can be expressed on different scales, such as meters, or feet and inches, so detail vision can be expressed on different scales. The most common scale is the visual acuity scale, introduced by Snellen (1862) [5], based on a proposal from Donders (1861).

More recently, other scales have been introduced, such as the logMAR scale. It is important to understand that the visual acuity scale and the logMAR scale are just different scales on which the same concept of detail vision can be expressed. Although many use it that way, the term visual acuity is not a substitute for the concept of detail vision. The often-seen expression “visual acuity as logMAR” is as wrong as “inches expressed as meters”; The proper expressions are detail vision expressed as visual acuity and detail vision expressed as logMAR.

More details about this topic can be found in Appendix A.

### 2.2. Labels for Detail Vision

Letter chart measurements are easy and straightforward as a measure of *How the EYE functions*. Most practitioners, however, have only a vague idea about what various values mean in terms of *How the PERSON functions.* This has led to confusing definitions of terms like ‘low vision’ and ‘blindness’.

**Figure 6:**
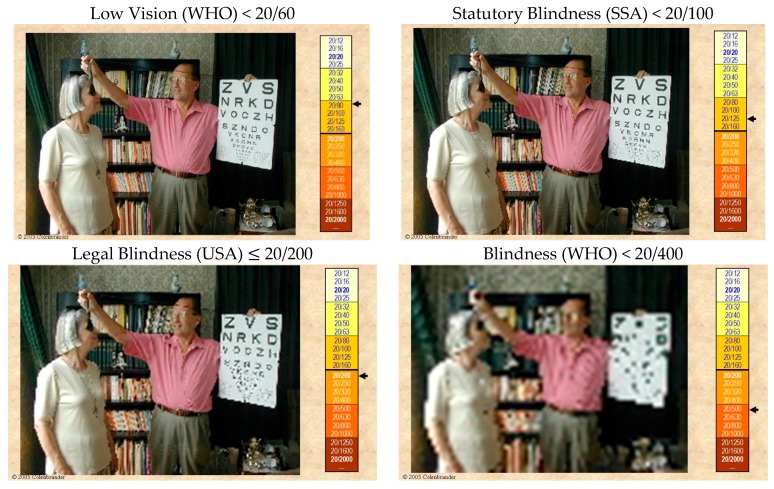
Confusing terminology.

The pictures in Figure 6 are chosen from a series of progressively blurred images (Appendix B). Since the portable letter chart is part of the picture, we can determine the visual acuity level that each picture represents.

The first picture shows the visual acuity level (<20/60) that is labeled as “Low Vision” by the World Health Organization (WHO). Although the bottom lines of the letter chart cannot be read, the appearance of the room is near normal. The next picture shows what the Social Security Administration (SSA) in the US considers “Statutory Blindness” (<20/100). It clearly is far from actual blindness. Other US programs use a different definition, called “Legal Blindness” (≤20/200), and the WHO does not consider persons “Blind” until they have reached the status of the last picture (<20/400). At that level, reading even the special letter chart is not possible, but one can still easily navigate the room. Even this level of visual impairment does not constitute actual blindness.

The widespread use of the word blindness is unfortunate, since it emphasizes what has been lost, rather than the abilities that remain. Also, the term cannot be used with modifiers: one is either blind or sighted; one cannot be “a little bit blind”. This negative emphasis and the black-and-white thinking have hampered the acceptance of vision rehabilitation. We should prefer terms such as “vision loss” or “visual impairment” that can be used with modifiers from mild and moderate to severe and profound and finally to total vision loss.

Although “legal blindness” (USA) and “severe vision loss” (ICD-9-CM) have the same definition, there is a big difference between telling patients “*You are now legally blind*” and telling them “*You have a severe visual impairment*”. The first statement might be followed by “*I am sorry, there is nothing more we can do for your eyes*”; the second statement leads to the question “*What can be done to help you cope with this problem?”*

### 2.3. Remediation for Loss of Detail Vision

Since visual acuity values are based on the amount of magnification needed to reach standard performance, it follows that magnification is the main countermeasure to visual acuity loss. This is expressed in Kestenbaum’s rule, which states that the minimum magnification required is the reciprocal of the visual acuity value. When actually prescribing magnification, usually somewhat more is required to account for the difference between threshold letter chart performance and comfortable reading performance.

There is a wide variety of magnification devices. We will only mention the main categories.

**Figure 7:**
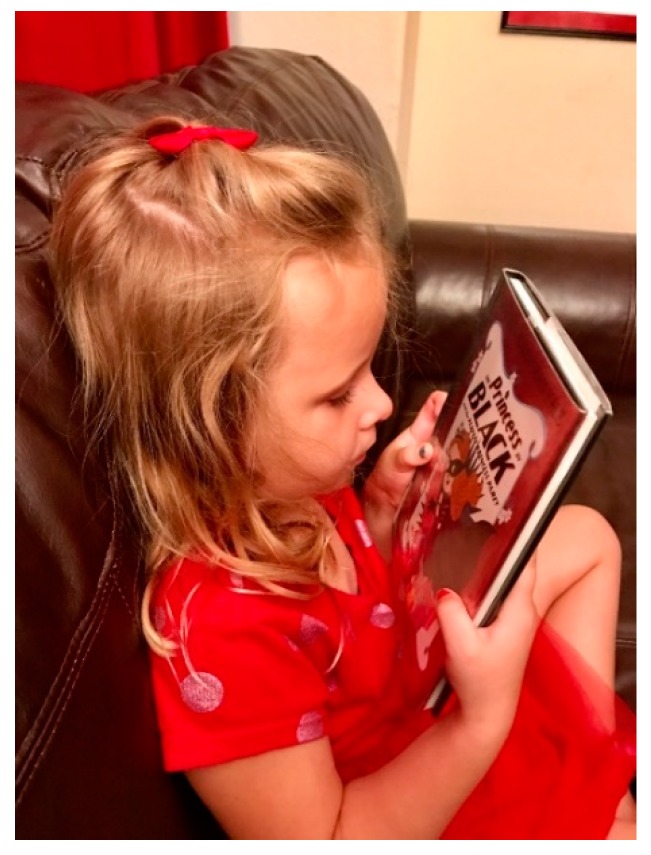
Child, using accommodation.

**Figure 8:**
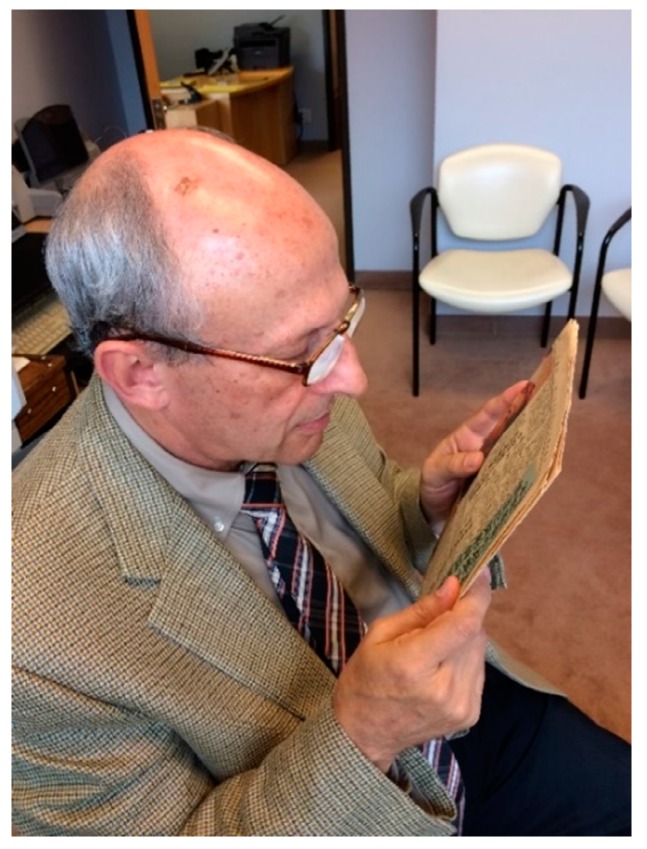
Adult, using reading glasses.

*Bringing print* (or other objects) *closer* is the simplest solution. Children with ample accommodation can do this effortlessly whilst seniors need reading glasses (Figure 7 and Figure 8).

**Figure 9:**
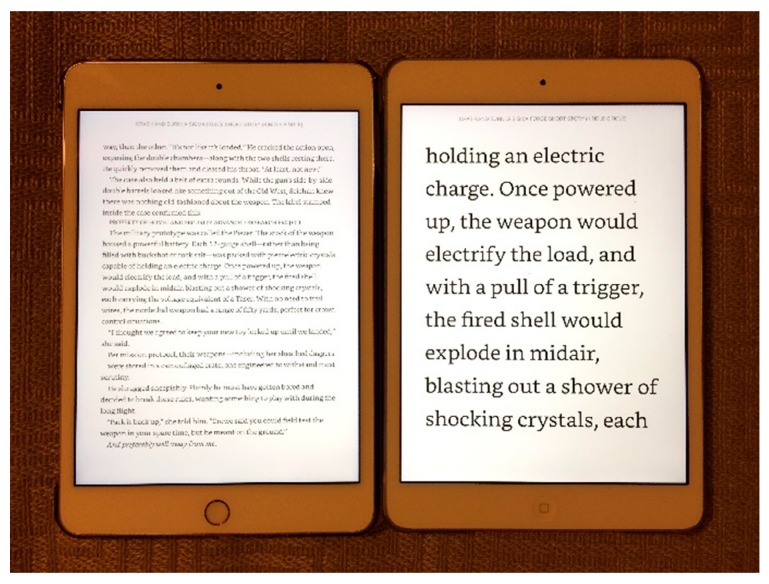
Tablets provide an easy way to produce large print.

**Figure 10:**
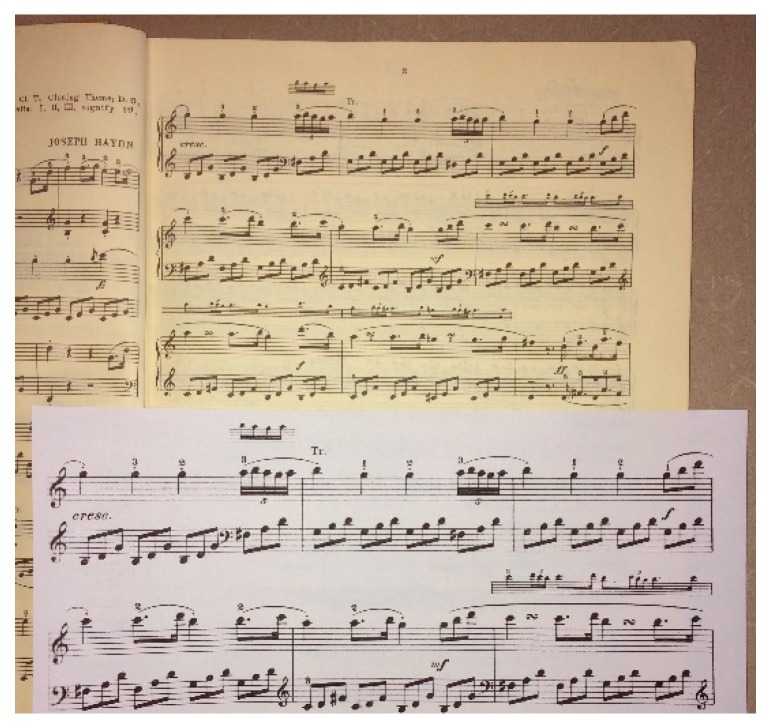
Music can be enlarged on a standard copier.

An alternative is to make the print (or object) *physically larger*. Examples are shown in Figure 9 and Figure 10.

**Figure 11:**
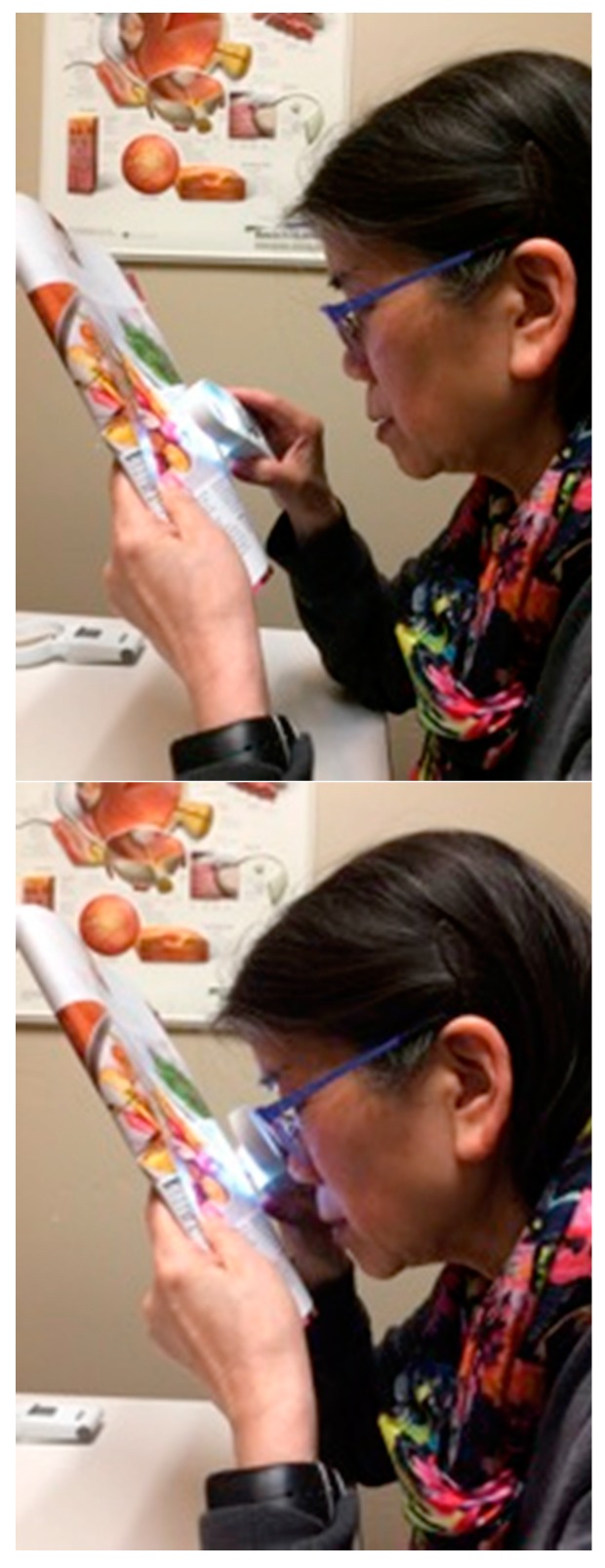
Handheld magnifiers.

**Figure 12:**
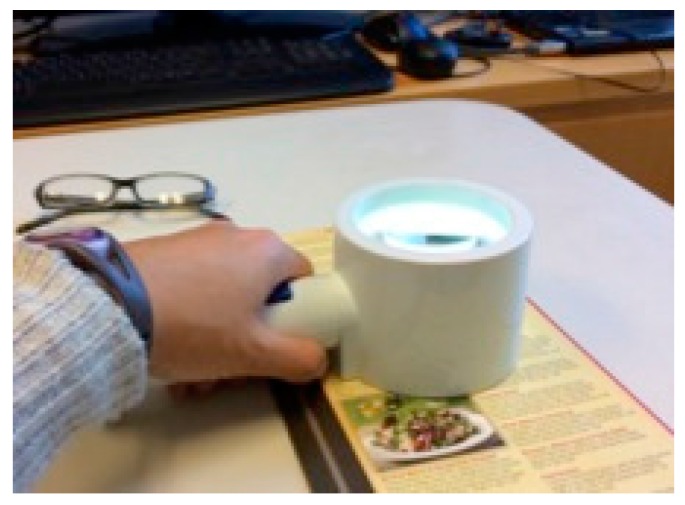
Stand magnifier with illumination.

Another alternative is the use of optical magnifiers, which make the print *appear larger*. They are available in a variety of powers, with or without built-in illumination (Figure 11 and Figure 12). They inevitably have a limited field of view, and higher magnification means an even more restricted field of view. The amount of magnification is fixed for each particular magnifier and has to be chosen in light of the patient’s particular needs.

**Figure 13:**
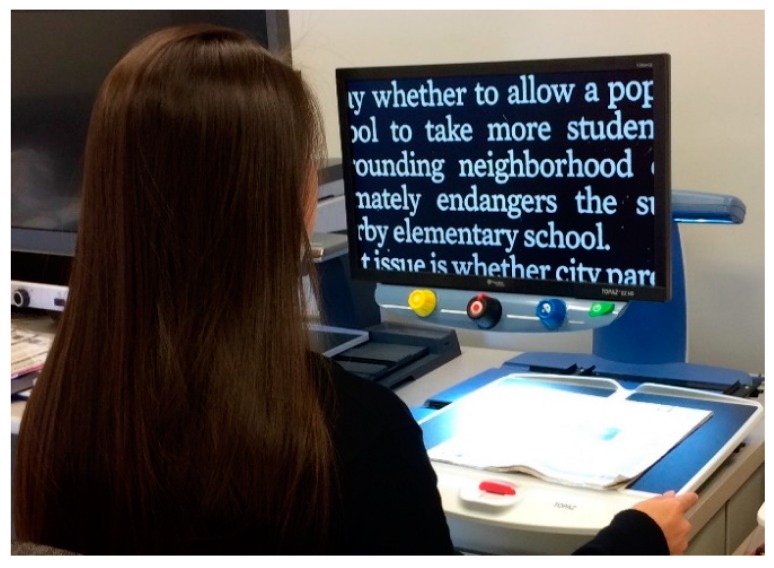
Desk-based electronic magnifier.

**Figure 14:**
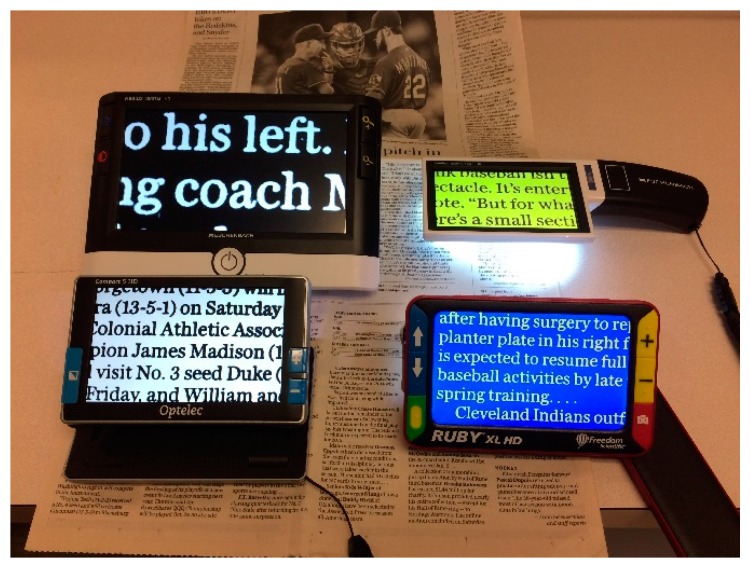
Handheld electronic magnifiers.

The newest solution is *electronic magnification*. These devices replace the original object with an enlarged image on a computer screen (Figure 13 and Figure 14).

They are more expensive, but also have significant additional advantages. The field of view depends on the size of the screen, rather than on the magnification. The amount of magnification can be varied, so the viewing distance can be increased by increasing the magnification. This allows better binocular viewing, even at high magnification, and improved ergonomics. Electronic magnifiers are available in desk mounted models with large screens, and in portable models with smaller screens. Larger screens can display more text or higher magnification; smaller screens are more portable. The screens can also provide enhanced or reverse contrast, or the use of colors, which is advantageous for some patients.

It is the task of vision rehabilitation professionals to help patients select the most appropriate magnification mode for each activity. Different solutions may be best for different tasks. Reading large amounts of text (text books for college students, contracts for lawyers) may benefit from a large screen, desk-mounted electronic magnifier. Reading price tags in a store may benefit from the portability of a simple optical magnifier.

### 2.4. Vision Substitution

An entirely different group of solutions is to replace the use of vision with the use of other senses. This includes the use of a cane to sense the depth of a step, use of a human guide in a crowded environment and the use of talking devices or a human reader for textual materials. We will not cover these options in this paper.

## 3. Surround Vision

### 3.1. The Nature of Surround Vision

That normal letter chart acuity alone is not sufficient for normal vision can easily be shown by looking through a narrow cardboard tube. One might still be able to read the entire letter chart, but navigating the room would be impossible. The need for vision beyond the central 1-degree area is captured under the heading surround vision.

The term surround vision is not as familiar as the term peripheral vision. We use the term surround vision because the term peripheral only refers to retinal topography. The term surround refers to how this aspect of vision is used. When reading, it refers to the area immediately surrounding fixation, which usually is still in the so-called central field. When moving around, it refers to the perception of the environment surrounding us.

The most important way in which surround vision is different from detail vision is how it is processed in the brain. In the brain, detail vision, which is used for the conscious identification of objects that surround us, is processed through the ventral stream. It allows us to recognize those objects by connecting to large memory banks that store information about all objects we have ever seen.

Surround vision, on the other hand, is processed through the dorsal stream in the brain. It constantly monitors our environment to avoid bumping into people or objects without the need to pay conscious attention to each object. It provides direct connections to motor systems, for the execution of body movements when walking, for arm and hand movements when reaching and grasping, and for eye movements when reading. It is processed autonomously and pre-attentively. Its activity is comparable to the way most body systems are controlled and regulated autonomously, without ever reaching consciousness.

A second function of surround vision is to send an occasional alert to the oculomotor system when it encounters a “salient” stimulus. This alert causes a reflex-like fixation movement so that the central system can be used to consciously recognize the object. This occasional alerting function is the best-known function of surround vision, but it could not exist without the continuous monitoring function.

The fact that most peripheral visual information is processed autonomously, sub-consciously and pre-attentively, and does not reach conscious awareness, explains why patients rarely complain about loss of surround vision. This is as true for loss of peripheral vision, which is important for mobility, as it is for scotomata in the central field, which are important for reading.

At this point, one should recognize that the terms central and peripheral refer to the anatomy of the retina; the terms ventral and dorsal stream refer to the anatomy of the brain. They are not identical. When taking in a panorama, the recognition system (ventral stream) takes in a fair amount of peripheral information. When reading, the oculomotor system (dorsal stream) guides eye movements, based on information from the central field, while the ventral stream processes that same information to understand the content. Thus, information from the same retinal receptors regularly ends up in both processing streams. This is just one example of the fact that the brain uses massively parallel processing.

For single-letter recognition a 1-degree visual field suffices, but for reading fluency it is not enough. Reading fluency requires the recognition of words, rather than individual letters. It also requires smooth eye movements from word to word. Both require a larger functional area, so that reading fluency not only depends on good detail vision, as measured by visual acuity, but also on an intact area around fixation. When defects in this area interfere with reading fluency, we speak of “scotoma interference”.

Therefore, when documenting the progress of macular degeneration, a reading test will give information about a larger retinal area than a simple letter chart test. A test with a Mixed Contrast chart (see under contrast vision) may be even more sensitive.

Scotoma interference can not only affect letter and word recognition, it can also affect the efficiency of reading saccades. It has been shown that eye movement training, separate from word recognition training, can improve reading fluency [6].

**Figure 15:**
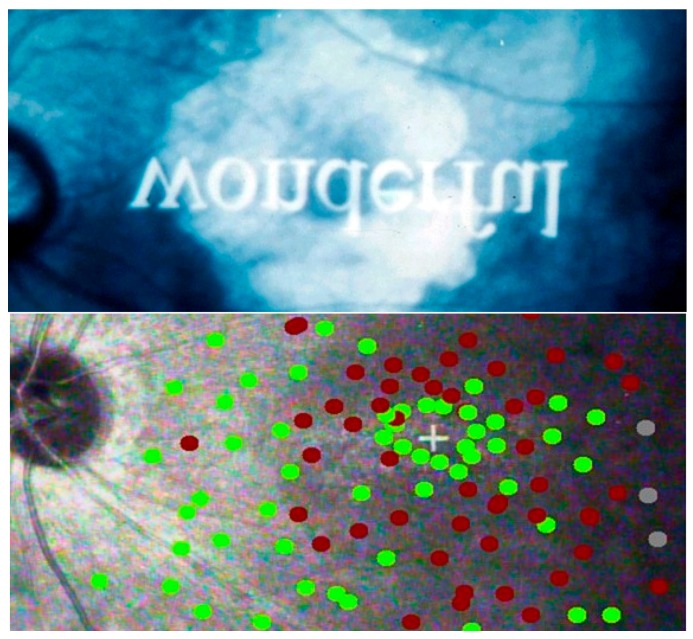
Ring scotoma Top: recognizing letters, but not words. Bottom: Micro-perimetry. Green = points seen; red = points missed. + = fixation.

Figure 15 shows how a ring scotoma can allow the recognition of a single letter, but not of a word. It also shows how situations like these can be assessed through micro-perimetry, which can provide fine detail by presenting stimuli directly on the retinal image. The second image shows the amount of detail that can be provided by SLO micro-perimetry. Regular perimetry, as used for more peripheral defects, cannot provide this level of detail.

When the fovea itself is no longer functional, the situation becomes more complex. In this case, the brain must select a pseudo-fovea, or Preferred Retinal Locus (PRL), to serve as the center of fixation. Usually, this point is located at the edge of the scotoma, as close as possible to the former fovea, since retinal resolution decreases with increasing distance from the fovea.

### 3.2. Remediation for Surround Vision Loss

We have seen that there are many devices that can alleviate the loss of detail vision. Unfortunately, loss of surround vision cannot be ameliorated with devices. Image magnification or image relocation (prisms) disrupts the coordination between the visual and the haptic appearance of the world, which is needed for good mobility. We know that for some people even the slight prismatic effect of bifocals can be disruptive and can make them stumble.

Because of this, use of optical devices is best when seated or standing; walking with them is not recommended. The key remediation procedure for surround vision loss, therefore, has to be *patient education*, so that patients understand the nature of their condition and how to deal with it.

The first item that patient education should deal with is patient expectations. Patients with AMD, are often worried because they have been told that their condition may worsen to a point where they are declared “legally blind”. They should be told explicitly that the very term “macular” degeneration denotes that the condition is limited to the macular area and will not affect their peripheral vision; so, they will always be able to navigate, even if their ability to see details may require more magnification in the future.

They should also be told that “legal blindness” is a legal term without any medical significance. It denotes that they are eligible for certain benefits; it does not denote that they are blind, or even that they are at risk of becoming blind (Figure 6).

Other consequences of vision loss should also be mentioned. Charles Bonnet syndrome can be so frightening that patients do not dare to mention it, unless prompted.

Patients with peripheral loss (glaucoma) need to be told that they need more scanning. For patients with scotomata in the central field (AMD), the configuration of the blind spots in the central field will determine how well they can read or perform other detailed tasks. Even if micro-perimetry (Figure 15) is not available, the position of blind spots can be ascertained by having the patient look at a spot on the wall (e.g., a doorknob), while presenting stimuli with a laser pointer. After the clinician has done this, it is useful to let the patients themselves move the laser pointer, so that they get direct proprioceptive feedback about their own blind spots.

In some cases, the foveal area is spared as an island with near-normal visual acuity. In these cases, the size of the central island will determine how useful it is. For some tasks, the central island may be adequate. If the island is very small, it may be advantageous to use a larger, more peripheral area for certain tasks, possibly with magnification to compensate for the reduced acuity. It is the task of the rehabilitation professional to help the patient in figuring out the best solution.

In other patients, the foveal area is spared, but with reduced acuity. Today, this is seen more commonly, since anti-VEGF therapy has replaced laser coagulation for AMD [7]. These patients can be helped with magnification, but do not need to adjust their fixation behavior.

In more advanced cases, where central fixation is no longer possible, the brain will select a Preferred Retinal Locus (PRL) as the new area for fixation. This requires not only magnification to compensate for reduced acuity, but also an adjustment of the oculomotor system to a new reference point.

It is important to recognize that the PRL is not a retinal feature, but the result of selection by the brain. It is not entirely clear how the brain selects the PRL, and whether the spontaneously chosen PRL is always the most effective one. The effectiveness of the brain’s choice depends not only on picking the area with the best visual acuity, but also on the ability to re-calibrate the oculomotor system to a new reference point.

**Figure 16:**
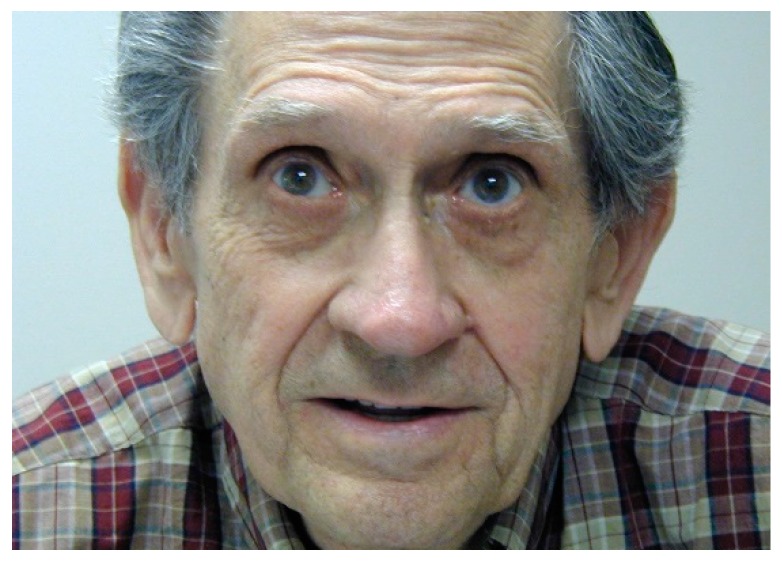
Viewing eccentrically. This man stated he was looking straight ahead into the camera.

As patients develop an eccentric PRL, it may appear as if they are not looking at the object of regard or at their conversation partner (Figure 16). Patients should be warned about this. Some may choose to alleviate the social awkwardness by occasionally looking at the partner, even though that will make the partner’s face disappear.

It has been shown that it is possible to change fixation from the original PRL to a Trained Retinal Locus (TRL), but opinions about the desirability differ. Some suggest that it is not worth the effort. Others have suggested that patients should be trained to choose the point of highest retinal sensitivity, or the point of highest resolution, or the point closest to the prior fovea. Still, others suggest that a point below the scotoma in the visual field (above the scar in the retina) will be optimal because it provides the widest horizontal area for word recognition. There are no definitive studies since training methods and the extent to which such training is offered and covered by insurance vary for different countries. Modern micro-perimeters may offer training sequences. Old fashioned paper and pencil tests for additional training at home also exist.

It is possible that a patient has more than one PRL, e.g., one for high illumination and another one for low illumination. How the selection of a PRL proceeds under binocular viewing conditions is also unknown, since all current diagnostic equipment is limited to monocular use.

## 4. Contrast Vision and Illumination

### 4.1. The nature of contrast vision

The third area of interest is contrast and illumination. Here even more questions about the exact mechanisms exist.

The fact that different charts are used for testing visual acuity and for testing contrast sensitivity, has led many to believe that contrast sensitivity is a third, independent parameter. This is not true. Contrast vision interacts with both detail and surround vision. Better contrast results in better visual acuity, and brighter visual field stimuli result in a wider isopter.

Contrast refers to brightness differences between adjacent areas. Contrast sensitivity is closely related to brightness sensitivity, but it is not the same. It is important to point out that the *contrast* of an object is independent of the illumination; however, how this contrast is perceived, depends on the *contrast sensitivity* of the eye, which is very dependent on illumination. At any point in time, the eye can distinguish a relative brightness range of about 1:100. However, light and dark adaptation can change the absolute position of this range enormously, so that the total range of perceivable brightness levels from a sunlit beach to a starlit meadow is at least 1:1,000,000. Some of this change happens very quickly (e.g., when driving into a tunnel), the remainder takes time. Complete dark adaptation takes at least 30 min. In patients with AMD, the speed of dark adaptation is often reduced.

The interpretation of contrast measurements is complicated by this interaction of contrast sensitivity and fast light-dark adaptation. It is also complicated by the fact that contrast sensitivity is influenced by at least three groups of factors: optical (pupil size), receptor-based (availability and use of visual pigments), and neural (gain control in the inner retina). AMD and glaucoma are both characterized by contrast losses, but the mechanisms must be different, since AMD is an outer-retinal disorder, and glaucoma is an inner-retinal disorder. This difference may be a reason why, when using electronic magnifiers, glaucoma patients seem to prefer reverse contrast more often than AMD patients do.

Gathering more information about contrast vision is further hindered by the fact that most practitioners don’t measure contrast vision because it does not provide actionable information for clinical decisions. This, however, may be a vicious circle; if it is not measured, its clinical significance cannot be assessed. Several studies have suggested that low contrast losses may be a more sensitive detector of early disease than high contrast letter chart losses.

For the patient, contrast deficits are definitely important, since they can have significant consequences, especially for mobility and safety. Warning a patient to be alert and careful about low contrast situations, such as stepping down from a curb, could prevent a broken hip. Not serving white rice on a white plate on a white table cloth can avoid unnecessary embarrassment.

It is reasonable to assume that retinal units are not just alive or dead, but that they can show various levels of sensitivity. Units that are marginal may not respond at low light levels, but become active when the light level is increased. This explains why AMD patients generally benefit from increased light, as well as from increased contrast. It also suggests that relative losses at supra-threshold levels (using the Mixed Contrast cards) may be a more sensitive indicator of early disease stages than absolute losses at the threshold level.

### 4.2. Assessment of Contrast Vision

Most clinicians do not measure contrast sensitivity in routine practice because it is an extra test that does not contribute to clinical decision making. Above, we have argued that it may contribute to early diagnosis. This is definitely important for a treatable disease such as glaucoma and will become important for AMD if early treatments are developed in the future.

**Figure 17:**
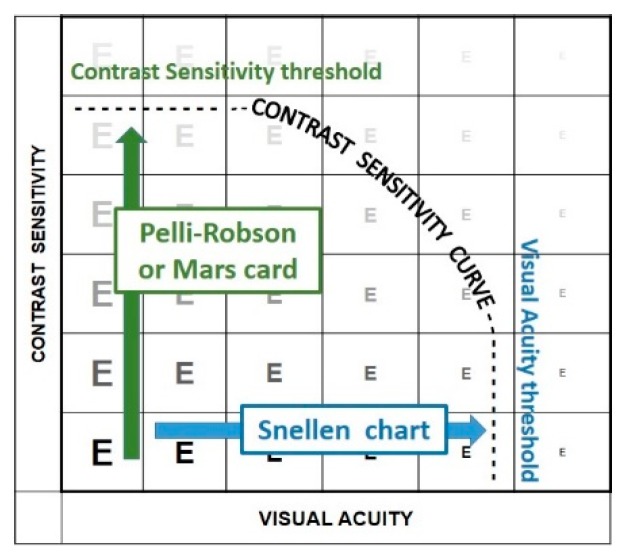
Threshold measurements.

**Figure 18:**
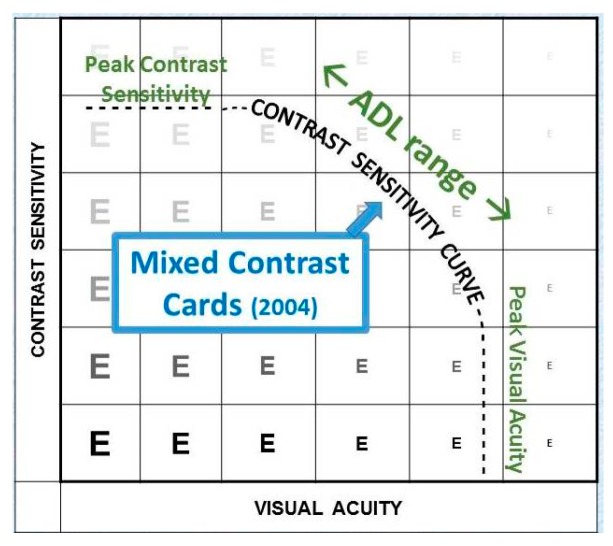
Supra-threshold measurements.

The most common contrast test is the Pelli-Robson chart. This test determines the just perceptible threshold contrast (Figure 17). Threshold tests are preferred for scientific measurements since thresholds are well defined and easily reproduced. However, few daily tasks involve threshold contrast (Figure 18).

**Figure 19:**
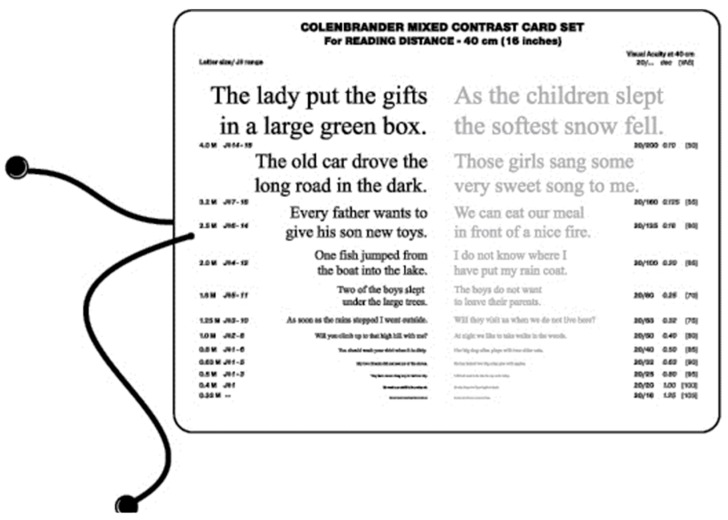
Mixed Contrast reading card.

The Mixed Contrast™ cards [8] (Figure 19) compare visual acuity with high contrast to visual acuity with moderate contrast (20% Weber). This supra-threshold low contrast measurement is more realistic for activities of daily living; it tests the slope (HC-LC difference) of the contrast sensitivity curve, rather than its endpoints. The test presents HC and LC text side-by-side on one handheld chart, which makes the test fast and allows easy comparison. The result is readily understandable for the patient, which avoids long explanations. The HC-LC difference is largely independent of the visual acuity level (the Pelli-Robson score is not); one possible explanation is that the visual acuity score reflects the density of the active receptor network, while the HC-LC difference reflects the sensitivity of the individual elements.

### 4.3. Remediation of Reduced Contrast Vision

As with losses of surround vision, most patients are not aware of contrast problems as such. The layout of the Mixed Contrast cards helps them to understand its effects and makes warnings about low contrast conditions more intuitive. Seniors may want to carry a cane, just to feel the height of a step. Not warning them, may result in a fall and a broken hip.

Better illumination is important. This includes better task lighting, as well as better overall illumination. Since dark adaptation may be delayed, patients should expect this effect when going into a theater. Wear sunglasses outside, even on an overcast day; take them off once inside, and part of the dark adaptation will have happened already.

Night driving will be a special hazard that patients should be warned about.

Some practitioners forego accurate refraction once the high-contrast visual acuity is reduced. However, blurred contours in the retinal image will hinder edge detection. Therefore, sharpening the retinal image by providing the best refractive correction is important, especially when the visual acuity is reduced.

Contrast cannot be increased with a device and contrast detection cannot be trained. Therefore, beyond advising caution, environmental modifications are the most important interventions. Provide more contrast wherever possible, do not serve milk in a Styrofoam cup, use a contrasting placemat, when serving white rice on a white plate on a white tablecloth.

Electronic magnifiers are advantageous (Figure 13 and Figure 14) because they can increase the contrast.

## 5. Conclusions

The care of patients with AMD involves more than the care of the retinal condition alone. Comprehensive rehabilitation requires teamwork. Retinal specialists will remain central to the treatment of AMD and should seek cooperation with other team members, who can provide the components for which they were not trained.

Areas of special interest include:

- **Detail vision**—Consider possible rehabilitation needs of anyone with <20/40 [1].
- **Surround vision**—Requires patient education, rather than devices.
- **Contrast vision**—Requires patient education and environmental modifications.

## Figures and Tables

**Figure A1:**
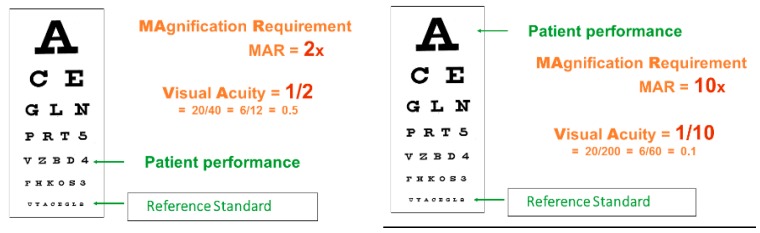
Visual acuity measurement.

**Figure A2:**
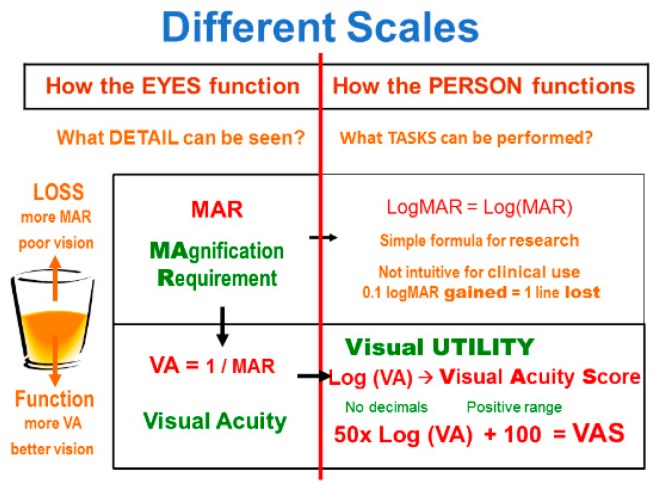
Scales for detail vision.

**Figure A3:**
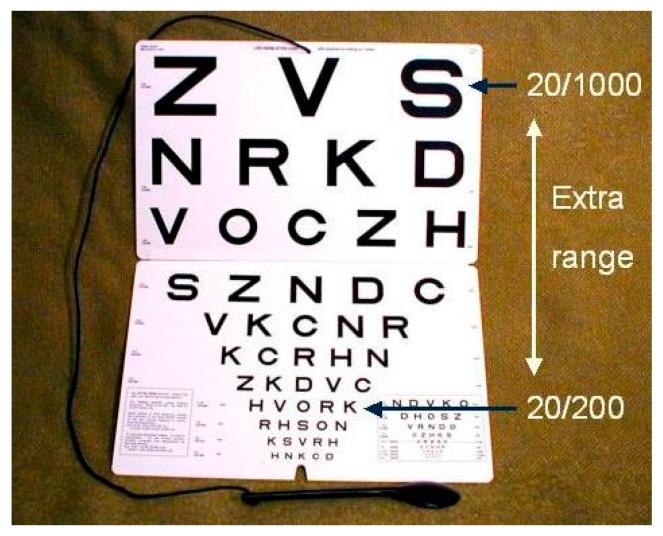
Extended range of a 1-m chart. The chart extends the range by seven lines.

**Figure A4:**
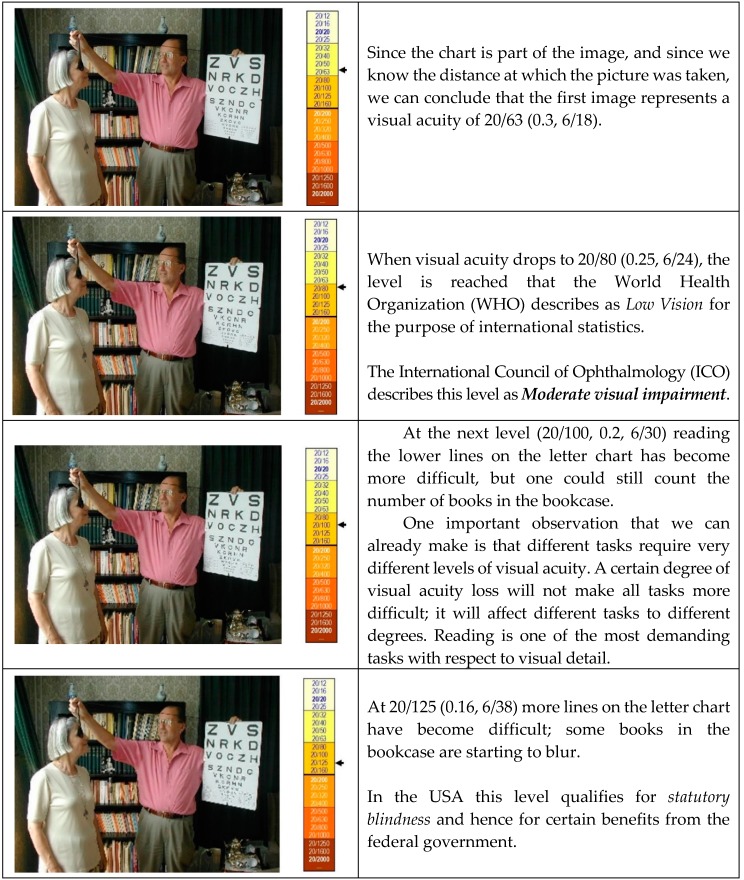

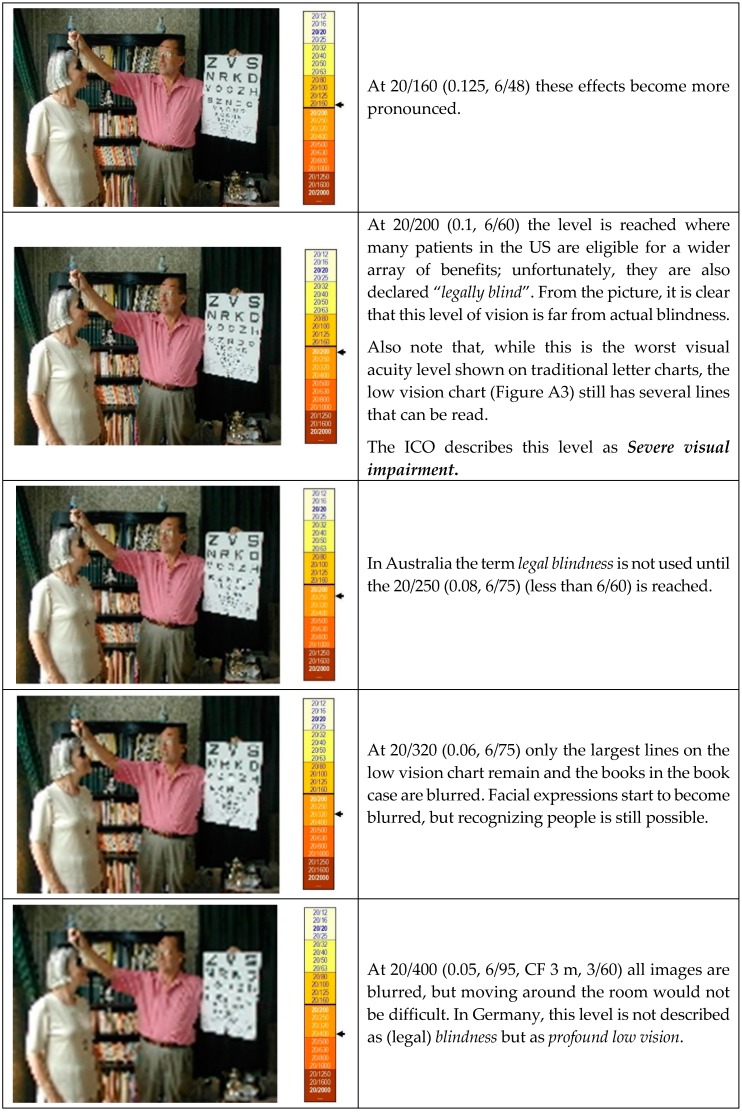

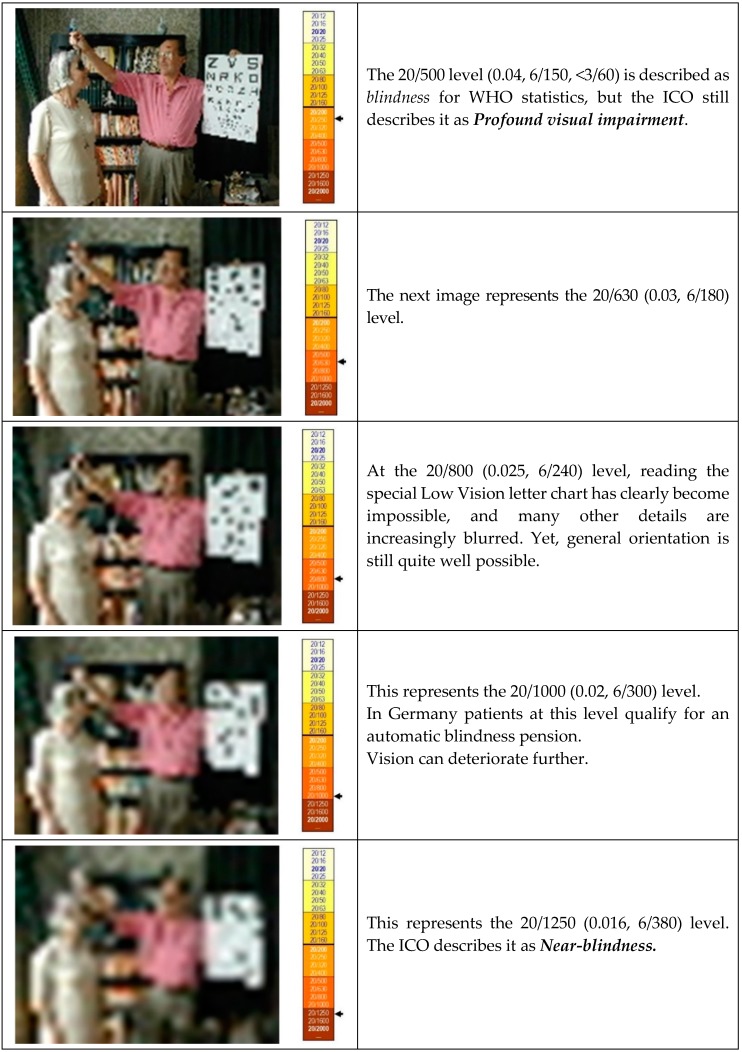

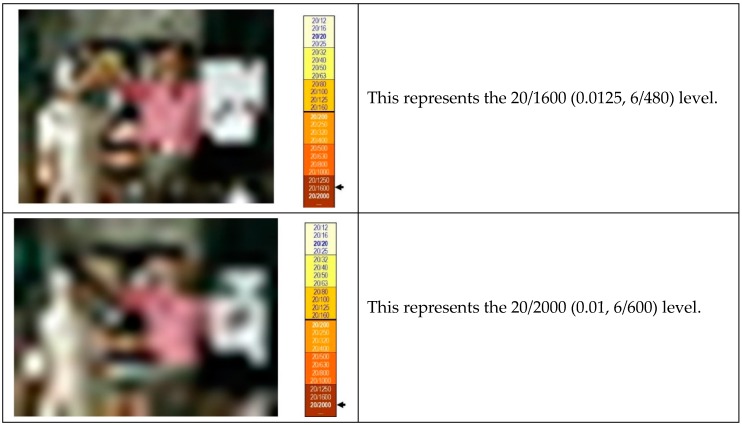
Visual acuity values at different levels.

## Appendix A. Measuring Detail Vision

### Appendix A.1. Letter Chart Acuity

The most common measure of detail vision is visual acuity, as introduced by Snellen in 1862 [5]. Snellen defined a standard observer as someone who could recognize one of his “optotypes” when it subtends 5 min of arc. He described this print size as one “easily recognized” by a normal eye, and labeled it as “20/20”.

When a patient needs letters that are 2× as big, visual acuity is said to be 1/2. If 5× magnification is needed, visual acuity is 1/5; for 10× magnification, the visual acuity value is 1/10, etc. (Figure A1). For use in the US the numerator of these fractions is commonly changed to “20”, resulting in notations such as 20/20, 20/40, 20/100, 20/200, etc.

Snellen used printed charts, which were calibrated for specific distances. Using his notation, the numerator indicated the exact test distance; the denominator, the exact letter size. His original charts (1862) were used at 20 Parisian feet (at the time more than 20 different feet were used in different parts of Europe). When the Treaty of the meter was signed (1875), he switched to charts for 5 m or 6 m. Recognizing that 5/10 and 6/12 indicate the same visual angle, Monoyer [9] (1875) proposed to use decimal fractions instead. In the US, the original 20/20 notation was maintained. Elsewhere, metric notation (5/5, 6/6) was used. While most of Europe gradually adopted the decimal notation, Britain maintained the 6/6 notation (20 ft = 6 m).

Today, the numerator is commonly standardized, and has no relation to the actual test distance anymore. For printed charts, the test distance must be adjusted to the chart design. For projector charts and computer displays, the display sizes can be adjusted to whatever distance is used. Therefore, the same numerator is used for distance and for near vision. Instead of speaking of Snellen fractions, we now speak of “Snellen equivalents”.

### Appendix A.2. Measurement Scales

When using a letter chart, the quantity measured is the size of the letters that is needed to achieve threshold performance. That size is then compared to the size just recognized by a standard observer. This yields the MAgnification Requirement or MAR value (Figure A2). The MAR value does not specify the reason why magnification is needed. When limiting the discussion to the optical system of the eye, MAR is often interpreted as Minimum Angle of Resolution.

The MAR value is a measure of loss, since a higher value indicates poorer vision. For rehabilitation purposes, we would rather have a function scale. We can achieve this by inverting the value: VA = 1/MAR. This is the definition of visual acuity. Converted to MAR = 1/VA, it is also known as Kestenbaum’s rule.

MAR and VA are thus scales of “*How the EYE functions*”. They indicate what detail can be seen. For rehabilitation purposes, we would like a measure of “*How the PERSON functions*”, by asking what tasks can be performed. Weber-Fechner’s law tells us that we can approximate that value by taking the logarithm of the previous value. We may call that concept “visual utility”, reflecting the *usefulness* of vision, to distinguish it from the “visual acuity” scale, which reflects the *sharpness* of the retinal image.

Just taking log(VA) gives us a scale with inconvenient negative numbers. Multiplying by 50 avoids decimal values and adding 100 brings the scale to the positive range; this results in the “visual acuity score” (VAS). This score was adopted by the International Council of Ophthalmology (2002) [10] and by the American Medical Association [11,12] (2001) for calculation of disability estimates.

The visual acuity score is intuitive, since it places 20/20 at 100 (or 100%), and because it increases by one point for every letter read correctly on a chart in ETDRS format (see below). The scale is not truncated at 100; “normal” detail vision (>20/20) results in scale values that are greater than 100.

Another logarithmic score simply takes the logarithm of MAR. This results in the logMAR scale. Because of its mathematical simplicity, this scale is often used in scientific papers. For clinical use, however, it is less intuitive, since it is a scale of loss (higher values indicate poorer vision), because of its decimal values, and because “normal” detail vision results in negative logMAR values.

### Appendix A.3. Logarithmic Progression

Clinicians have often described changes in functional vision as “lines gained” or “lines lost”. That notation is valid only when all steps between lines are equal. That requires a logarithmic progression. The use of a strict logarithmic progression, instead of Snellen’s partially logarithmic progression, was first proposed by John Green in 1868 [13] after he returned from his studies in Europe, where he also worked with Snellen. He was ahead of his time. Over the years, his proposal was used by various others, but the strict logarithmic progression did not become generally recognized until a century later, when the National Eye Institute in the US used it to standardize visual acuity measurement for its various studies, the first of which was the Early Treatment of Diabetic Retinopathy Study (ETDRS) [14].

In addition to using a logarithmic progression, the ETDRS charts abandoned the traditional rectangular format with few large letters and many small ones. They adopted a triangular format with 5 proportionally spaced letters on each line [15].

### Appendix A.4. 1-m Chart

Snellen’s chart (Figure A1) and most subsequent charts, had no letters larger than 20/200. For worse detail vision, Snellen recommended changing the testing distance. Others just used ill-defined approximations, such as “count fingers” and “hand motions”.

The chart shown in Figure A3 combines the ETDRS principles with a 1-meter cord attached to the chart. This makes it simple to extend accurate measurement to the 1/50 (20/1000) level.

## Appendix B. Samples of Detail Vision

**Visual acuity values** are widely used to denote the quality of vision, but most people have very little idea of what kind of vision the various acuity values represent. The following images are meant to give an impression of the various levels.

The images were prepared by gradually reducing the number of pixels used for the image. Printed images may not have the same detail as the original PowerPoint slides. Standard printing or projection is not good enough to represent 20/20 (1.0, 6/6) visual acuity. With that level of visual acuity, it would be possible to read part of the insert on the bottom right corner of the chart.

At these last levels, mobility becomes a problem and one might bump into people or furniture. But even these levels do not represent total blindness.

When looking at these images, it is clear that vision loss can occur along a continuum of stages. As this happens the number of tasks that become difficult increases, but there is no obvious breakpoint where the quality of vision suddenly changes. Thus, the use of dichotomous descriptors, such as sighted/blind always remains arbitrary.

Such descriptors are used nevertheless. This is why it has been said that

**More people are blinded by definition than by any other cause.**
